# Mediating factors explain anxiety experienced by women with obesity during the Covid-19 pandemic

**DOI:** 10.1371/journal.pone.0295034

**Published:** 2023-12-20

**Authors:** Isabel Urdapilleta, Lionel El Khoury, Jean-Marc Catheline, Saadi Lahlou, Samuel Demarchi

**Affiliations:** 1 Department of Psychology, Laboratoire Cognitions Humaine et Artificielle (CHArt—EA 4004), Université Paris 8, Vincennes-Saint-Denis, France; 2 Digestive Surgery Department, Delafontaine Hospital, Saint-Denis, France; 3 Department of Psychological and Behavioural Science, London School of Economics and Political Science, London, United Kingdom; 4 Paris Institute for Advanced Study, Paris, France; St John’s University, UNITED STATES

## Abstract

The Covid-19 pandemic could be a source of great anxiety, especially for those at higher risk, such as women experiencing obesity. The aim of this study was to measure how some personal characteristics such as BMI (from underweight to class 3 obesity), bariatric surgery (yes or no), comorbidities, or age (as antecedent variables), and mediating factors impacted state anxiety during the Covid-19 Pandemic. Mediating factors were related to subjective knowledge or attitudes (e.g. interest or beliefs and practices around Covid-19, subjective health perception, and confidence in the government). French women (*N* = 532) were invited to take part in a voluntary online health survey during lockdown in Paris and its suburbs. Results showed that women with higher BMI had higher anxiety scores, primarily because they feel less healthy than other people. Secondly, the larger the body size of the participants was (BMI), the less they reported that information about Covid-19 held their attention. This lack of interest resulted in feelings of anxiety not being generated. Thirdly, the larger their body size was, the less confidence they had in the effectiveness of the proposed measures by the government and therefore, the more anxious they were. Finally, older age predicted higher interest in the pandemic, higher subjective health, and higher confidence in the government. Identifying obesity as a potential risk factor for anxiety disorders is crucial, but measuring the relationship between state anxiety and personal characteristics (e.g. BMI) requires considering mediating variables (e.g. subjective health perception). To reduce anxiety in women with obesity, it appears necessary to focus on psychological programs that can help them improve their perception of their health, as well as the confidence they may have in institutions, especially for younger women.

## Introduction

On 11 March 2020, the World Health Organization (WHO) [1] declared Covid-19, a disease caused by coronavirus SARS-CoV-2, a pandemic. Efforts to control and reduce coronavirus transmission rely on behavioural change. In France, the entire population was placed under lock-down. The outbreak generated drastic emotional, social, and economic changes. Furthermore, living with the Covid-19 pandemic has been an unprecedented crisis for people around the globe, with considerable impact on public mental health. The psychological conditions of individuals significantly deteriorated during the Covid-19 outbreak (see [2], for a Review), as the uncertainty and severity of this pandemic situation not only impact physical health, but also mental health.

A large number of studies are now being carried out on the mental health consequences of the current Covid-19 pandemic. Many of them have compared the level of various mental health indicators measured in cross-sectional surveys conducted on the general population [3], pregnant women [4], lesbian, gay, bisexual, and transgender adults [5], college students [6], university students [7] and healthcare professionals [8]. All of them exhibit a deterioration in mental health during lockdown. The WHO has also expressed its concern over the pandemic’s mental health and psycho-social consequences [9]. It speculates that new measures such as self-isolation and lock-down have affected people’s usual activities, routines, and livelihoods which may lead to an increase in loneliness and anxiety.

Covid-19 has not affected everyone equally. Obesity has been reported as a risk factor for severe form of coronavirus disease [10]. Data from France have shown a disproportionately higher prevalence of obesity in patients with Covid-19 admitted to Intensive Care Units (ICUs) compared with general population data [11]. Additionally, analyses from Lille University Hospital (France) have reported differences in BMI distribution in ICU patients with Covid-19 compared to ICU patients without Covid-19 [12]. Evidence was emerging that obesity related conditions seem to worsen the effect of the virus [13]. The French government’s advice for those with a BMI of at least 40 kg/m^2^ is to be particularly stringent in following social distancing measures: "There is a clear link between obesity and the risk of Covid-19 complications, both because of and independently of the associated pathologies" [14]. It is likely that this message has created confusion and anxiety for many people living with obesity because of the uncertainty about their risk and what actions they should take.

The aim of this article was to measure how some personal characteristics that are said to have impact on Covid-19 severity (e.g., BMI, bariatric surgery, comorbidities, and age) and mediating factors (e.g., subjective health) impact state anxiety during the Covid-19 Pandemic. Body size was measured by Body Mass Index, in women who have and have not undergone bariatric surgery. Evidence has shown that practices and belief of people in society play major roles in the emotional regulation of anxiety. It requires the acquisition of knowledge and a change of practice to reduce it [15, 16]. For this reason, beliefs and practices around Covid-19, the perceived effectiveness of the proposed measures by the government and the subjective health perception associated with Covid-19 of women with different body sizes and body size histories have also been studied, in order to better understand what representations drive their state anxiety during the Covid-19 pandemic. State anxiety is an unpleasant emotional state, usually temporary. This state is said to exist at a particular time, to have a particular level of intensity, and to be characterized by feelings of tension, apprehension, nervousness and worry [17].

### Anxiety in persons with obesity

It is common to think that obesity may lead to anxiety disorders through various pathways. However, the current evidence linking obesity and anxiety disorders in the scientific literature remains unclear. Some studies have identified an association between these two conditions while others have failed to observe a significant relationship [18]. In fact, prospective evidence for an effect of obesity on anxiety disorders is scarce and mixed [see 18, for a review].

Furthermore, results from meta-analyses and systematic reviews point to a positive association between anxiety disorders and obesity or overweightness [19, 21]. These results should be viewed with caution in relation those reported in [20] who performed a systematic literature search of epidemiological studies that provided a quantitative measure of association between obesity or any specific type of anxiety disorder. Authors [21] concluded that no clear-cut pattern of results related to the link between anxiety and BMI can be observed and "a majority of the studies were undermined by methodological limitations and the observed associations were weak and often not significant". The limitations that Gariepy et al. [21] faced, and which can explain the contradictory results, concern the kind of measures of anxiety because some of them deal with state anxiety or trait anxiety, others on somatic symptoms of anxiety as including tension, worry, fear, panic, difficulties in relaxing, and restlessness, etc., see [22], or the characteristics of the participants considered. As Haghighi et al., (p. 49) state "anxiety disorder is an umbrella term to describe a group of psychiatric disorders characterized by an exaggerated psychophysiological reaction to internal or external stimuli which are subjectively considered as threatening" [23].

Other variables may moderate the association between obesity and anxiety, including the degree of obesity. The weight status of a person with obesity (as measured by BMI) is not the only criterion to be considered: whether or not they have undergone obesity surgery is another. Albert et al. study finds a significant decrease in anxiety among severely obese individuals waiting for a surgical procedure [24]. Moreover, contrary to individuals with abnormal scores on psychological well-being from the general population during the pandemic [2, 25], candidates with obesity waiting for a surgical procedure displayed lower anxiety during social isolation. So, the relationship between obesity and anxiety disorders might differ among subgroups of the population. In this context, gender seems to be an important factor as some studies [26, 27] reported positive associations between obesity or being overweight and anxiety among adult women but not among men. In addition to gender, several studies reported younger age as a risk factor for anxiety problems with Covid-19 [28]. The presence of comorbid physical health problems like diabetes, cerebrovascular diseases, heart diseases and other chronic conditions as a risk factor was associated with anxiety during Covid-19 [29, 30].

Several other factors associated with the mental health impacts of Covid-19 have been reported. For example, Wang and colleagues [31] reported that a high level of confidence in doctors or government communication (for similar results obtained before the Covid-19 pandemic, see [32]), perceived likelihood of survival, personal protective measures and satisfaction with health communications minimized the risks of adverse mental health outcomes during Covid-19 [33]. Subjective health also seems an important factor for understanding anxiety during the Covid-19 pandemic. The study by Szabo et al. revealed that individuals who perceived their health to be below average considered Covid-19 more threatening than those who perceived themselves to be in good health [34].

Thus, the level of anxiety related to the Covid-19 epidemic (dependent variables) can be expected to be dependent on the characteristics of the participants (independent variables) but also on their representation of the situation (mediator variables), beliefs and practices. In short, it seems it is not just the "objective" risk that matters, but rather its subjective perception and the confidence of the subject in individual (and social) agency to fight it. But what are the actual influences of such mediating factors, and how can they be measured?

### Beliefs and practices: The concept of distance to object

The multifaceted nature of human representation affecting beliefs and practices needs to be analysed using methods and theoretical options that integrate this multiplicity of factors.

In the context of the Covid-19 pandemic, Distance to Object (DO) appears to be a useful concept for understanding the situation. This concept was developed within the context of the Social Representations Theory [35, 36], to study the role of psychosocial variables, and seems especially adapted to analyse reactions to a threat that can appear “close” or “distant”. DO describes the relationships that individuals and groups may develop with a social object [35, 37], and is made up of three components [38]: knowledge (i.e., more or less adequate identification of the social object); involvement (i.e., degrees of concern, through social participation, toward the social object); level of practice (i.e., behaviours regarding the social object). Knowledge refers to the more or less satisfactory identification of the object by the individual, a cognitive distance and familiarity. Involvement could be compared with the relationship the individual has with the object or the extent to which he or she feels concerned about it, or his or her positioning with regards to the object, some kind of emotional distance. Finally, the level of practice is associated with the practices (considered as behaviours) developed in relation with the social object, so a behavioural distance.

Note that while some authors empirically validated this theoretical proposition by demonstrating that DO can mobilize three components (level of practice; knowledge; involvement [37]), other authors [39] validated a five component model: involvement (with three sub-components: object valuation, i.e., a social object which is or not important for oneself; personal identification, i.e., one feels concerned or not by this social object; and perceived capacity for action, i.e., I think I can or cannot act on this social object), knowledge, and level of practice, all related to the social object under study. This five-component model makes it possible to account for all the dimensions linked to the object under study [40, 41].

The DO concept is based on the assumptions that every individual can be positioned with respect to his/her distance to a social object (in this study, the social object is Covid-19). Each position highlights the relationship that the individuals (or groups) have with the social object and how this relationship influences the beliefs or expectations developed around this object (in our case Covid-19). In other words, individuals who are "close" to an object (short distance to it, e.g. by direct continuous experience of interaction with it) are those who have a greater knowledge of this object, feel more involved with it, and develop more practices related to this object than those who only were exposed to hear-say.

DO has been used to study various social objects including culinary choices [37], cannabis [42], cocaine [38] and wine [41].

In order to study the beliefs and practices around Covid-19, we used the concept of Distance to the Object "Covid-19", measured by a specific scale (see Appendix A in S1 File). To complete this investigation, we also measured the participant’s confidence in the perceived effectiveness of the proposed measures by the government (confidence in government), changes in reported practices regarding Covid-19, and perceived subjective health status.

Hypotheses regarding the present study are available in Appendix B in S1 File.

## Material and method

### Participants

The study included 532 French women aged 18–91 (*M* = 43.1, *SD* = 13.5) who lived in Paris or the surrounding area (see Table 1).

**Table 1:** Participants characteristics.

Weight Status	BMI		Number	Bariatric Surgery		Age	
	*M*	*SD*		*No*	*Yes*	*M*	*SD*
UW	17.37	1.29	21	21		41.29	19.12
NW	21.80	1.73	180	154	26	40.29	14.64
OW	27.24	1.23	96	62	34	46.24	13.01
O1	32.67	1.31	78	34	44	46.40	11.96
O2	37.32	1.62	75	44	31	43.25	11.80
O3	47.57	8.16	82	61	21	42.78	11.09
Total	30.36	9.94	532	376	156	43.10	13.47

Note: UW (Underweight, BMI < 18.5); NW (Normal weight, 18.5 > BMI < 24.9); OW (Overweight, 25.0 > BMI < 29.9); O1 (Class 1 obesity, 30.0 > BMI < 34.9); O2 (Class 2 obesity, 35.0 > BMI < 39.9); O3 (Class 3 obesity, BMI 40.0 and above).

The exclusion criteria were age (only people over 18 years of age could take part in the study), not having fully completed the survey (six women) and gender (too few men responded and few of them (*n* = 13) had undergone obesity surgery).

Women who have undergone bariatric surgery were recruited through the hospital where they had their surgery, through a call for volunteers, and through snowball sampling. Data for women who have not undergone bariatric surgery were collected from women starting with a convenience subsample of university employees and social networks, and then using the snowball sampling method.

Participants were provided with an internet link to an online questionnaire. Each trial proceeded as follows. The participants first were advised that the study involved some questions about how people cope with Covid-19 and how they were coping with the current situation. It was stated that the online questionnaire included questions about thoughts, feelings, concerns, and personal situation. Then they read the Information Sheet and signed the Consent Form (required by the university’s Human Ethics Committee). The protocol was validated and approved by the Psychology and Behavioural Science Ethics Committee at the London School of Economics and Political Science (Houghton St, WC2A 2AE, London, UK). Participants were able to ask any questions they wished via a specific email address.

## Design and procedure

Participants were asked to complete a set of questionnaires and scales during the third wave of French restrictions (3 April to 3 May 2021). Participants had already experienced restrictions and this was the third time. Specifically, a 7 pm curfew was imposed on the entire population and a strict ten-kilometer limit was placed on travel from one place to another outside the home (except for essential reasons such as buying food, medical appointments, work). All so-called non-essential shops, bars, restaurants, cultural venues and schools were closed. Gatherings were prohibited. The first wave of restrictions had been introduced between 17 March—11 May 2020. These were the strictest, and only travel within 1-kilometer radius from home during a one-hour period was allowed. A travel certificate had to be filled in and numerous police checks were made. The second lock-down was between 30 October and 15 December 2020. The measures were the same as the first one except that schools were open (but not universities).

After reading the Information Sheet and signing the Consent Form, the participants were invited to begin the questionnaire. The questionnaire began with the State-Trait Anxiety Inventory Form Y (STAI-Y, [17, 42], French validation [43], 20 items. Internal consistency coefficients for the scale have ranged from .86 to .95 [17]. The total score varies from 20 to 80.

Secondly, participants were invited to complete the Distance to Object (DO) questionnaire on a 7-point scale (1 = *Strongly disagree*, 4 = *neutral*, 7 = *Strongly agree*). The objective was to measure their distance to Covid-19. Because DO was conceptualized as a component variable scale involvement, (with three sub-components: object valuation, personal identification and perceived capacity for action), knowledge and level of practices, specific questionnaires were developed to characterize individuals on these five dimensions (40 items, see Appendix A in S1 File).

Finally, confidence in the perceived effectiveness of the proposed measures by government (confidence in government, 10 items), change in reported practices (10 items) and subjective health (1 item) perception were assessed. For each item, people indicated their opinion on a seven-point scale.

Age (in years), height (in centimeters) and weight (in kilograms), bariatric surgery (yes/no), comorbidity (yes/no: chronic respiratory insufficiency,—chronic renal failure on dialysis, insulin dependent type 1 and type 2 diabetes, etc.) were also recorded.

## Data analysis

### Preliminary analysis

Data analysis was completed using GLM Jamovi version 1.8.1.0 jAMM. All the inferential analyses were interpreted regarding an alpha level of .05. Investigation of the social representation of Covid-19 requires some specific measures for DO. The DO measurement is specific to each study because it is based on items dedicated to the theme under investigation. As stated above, we created a specific questionnaire to measure each DO component (and sub-component) related to Covid-19. Thus, we first present an analysis of the validation of the measures concerning each component and sub-component of the DO (item sensitivity and reliability analysis; [44, 45]). Secondly, we conducted a principal component analysis (PCA) and a confirmatory factorial analysis (CFA) in order to test the multidimensional structure of the questionnaire (three, versus five-dimension questionnaire, i.e., sub dimensions, [46–49]). Thirdly, concerning other scales, item sensitivity and reliability analysis were processed [44]. See Appendix C in S1 File for results about Preliminary Analysis, and Appendix D in S1 File for descriptive statistics about each variable and Appendix E in S1 File for the final version of the questionnaire.

### Main analysis

Spearman’s *r* correlation coefficients (two-tailed) and MANOVA were calculated to determine the relationships between the variables (see Table 2).

**Table 2:** Spearman correlation matrix, descriptive statistics, and mean difference between bariatric and no bariatric surgery for the main continuous variables.

Variables	1	2	3	4	5	6	7	8	9	10	11
**1. Stay–Y**	-										
**2. Age**	.00	-									
**3. BMI** ^**a**^	.06	.13**	-								
**4. Subjective Health**	-.38***	.06	-.33***	-							
**5. Object Valuation**	.19***	.34***	-.08	-.05	-						
**6. Personal Identification**	.16***	.22***	.40***	-.32***	.21***	-					
**7. Subjective Knowledge**	.07	.17***	.13**	.08	.43***	.04	-				
**8. LoP**^**b**^ **Transportation**	.10*	.12**	.02	-.08	.20***	.17***	.30	-			
**9. LoP Prophylaxis**	.04	.31***	.12**	-05	.42***	.22***	.29***	.25***	-		
**10. Confidence in Government**	-.09*	.23***	-.12**	.18***	.35***	-.05	.29***	.01	.18***	-	
**11. Change in Practices**	-.01	.03	.12**	-.18***	.08	.14**	.03	.14***	.17***	-.08	-
***M***	55.14	43.1	30.36	4.55	5.19	4.73	3.74	4.23	4.79	4.59	3.45
***SD***	12.66	13.5	9.94	1.42	1.82	1.86	1.68	2.13	1.92	1.14	1.05
***M***_**No bariatric surgery**_	**55.47**	**41.74**	**29.41**	**4.55**	**5.18**	**4.59**	**3.84**	**4.05**	**4.70**	**4.71**	**3.35**
***SD***_**No bariatric surgery**_	**13.22**	**13.86**	**10.40**	**1.47**	**1.85**	**1.89**	**1.71**	**2.10**	**1.99**	**1.10**	**0.97**
***M***_**Bariatric surgery**_	**54.34**	**46.37**	**32.64**	**4.53**	**5.21**	**5.05**	**3.49**	**4.68**	**5.01**	**4.30**	**3.68**
***SD***_**Bariatric surgery**_	**11.21**	**11.89**	**8.34**	**1.32**	**1.77**	**1.73**	**1.59**	**2.14**	**1.74**	**1.18**	**1.22**
***M***_**diff**_^**c**^	1.13	-4.62***	-3.22***	0.03	-0.03	-0.45**	0.35*	-0.63**	-0.30	0.41*	-0.33*
***M***_**diff**_ **95% CI [*LL*, *UL*]**	[-1.24, 3.50]	[-7.11, -2.13]	[-5.06, -1.38]	[-0.24, 0.29]	[-0.37, 0.31]	[-0.80, -0.11]	[0.04, 0.67]	[-1.03, -0.24]	[-0.66, 0.05]	[0.20, 0.62]	[-0.53, -0.14]

Note: ^a^ Body Mass Index; ^b^ Level of Practice; ^c^ *M*_diff._ = *M*_No bariatric surgery_—*M*_Bariatric surgery_

LoP Transportation is the level of practice related to Covid-19 precautions in public transportation

LoP Prophylaxis is the level of practice related to measures taken to prevent Covid-19

*p < .05

**p < .01

***p < .001

The global regression model explained an important part of the variance, with *R*^2^_adjusted_ = .18, *F*(12, 518) = 10.96, *p <* .001. A hierarchical regression model showed that personal characteristic variables (BMI, bariatric surgery, comorbidities, and age) or attitudinal variables (subjective knowledge, personal identification, level of practice transportation, level of practice prophylaxis, confidence in government, and change in reported practices), all accounted for less than 1% of the variance. Subjective health, by itself, explained 14% of the variance, whereas object valuation accounted for 3% of the variance.

**Fig 1:**
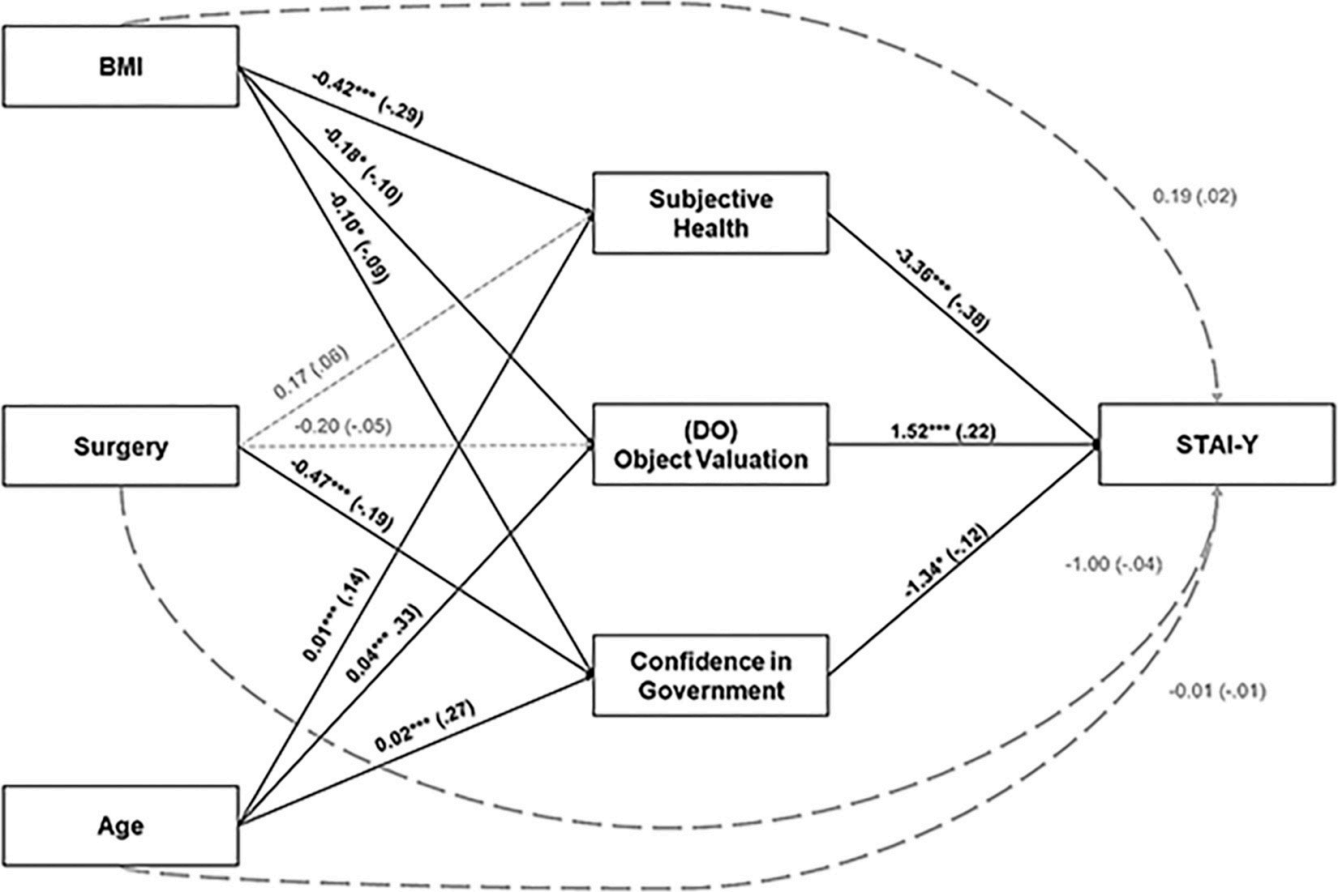
Regression coefficients *b* and (β) for the relationship between BMI, surgery, age, and anxiety (STAI-Y) as mediated by subjective health, object valuation, and confidence in government. Bold full black lines indicate significant results. Small, dashed lines indicate non-significant effect. Large dashed grey lines indicate non-significant direct paths. *p < .05, ** *p* < .01, *** *p* < .001.

Hypothesis 1c (see Appendix B in S1 File) was not tested since the perceived capacity of action dimension was removed according to the confirmatory factorial analysis (see the above CFA for the DO). The different test statistics for a hypothesis are displayed in Fig 1, except for some statistics that cannot be displayed in the figure (e.g., indirect components) reported in the text. According to hypothesis 1a, the closer participants get to object valuation (i.e., the more they think that the Covid-19 pandemic is important to them), the more they experience anxiety. We also observed that high scores on both confidence in government (hypothesis 1f) and subjective health decreased anxiety (hypothesis 1h). However, hypotheses 1b, 1d, 1e, 1g, 2a, 2b, and 2d did not reach significance level. Moreover, contrary to our expectations, hypothesis 2c showed that women with comorbidities exhibited lower anxiety scores, *b* = -3.07, β = .12, *p* = .011.

Mediation analyses (Hypothesis 3) were conducted to assess the mediating effects of Subjective Health, the five DO dimensions (i.e., object valuation, personal identification, subjective knowledge, LoP Transportation, LoP Prophylaxis), confidence in government and change in reported practices (mediator variables) on STAI-Y (dependent variables). BMI, comorbidity bariatric surgery and age were the antecedent variables. We only present the results of significant mediations (Fig 1). The order of presentation follows the order of the antecedent variables as mentioned above.

The total effect of BMI on anxiety (STAI-Y) was significant, *b* = 1.41, β = .11, *p* = .013. This was a full mediation effect, since no direct effect of BMI on anxiety was observed. This full mediation was mediated through subjective health and object valuation. Concerning subjective health as a mediator between BMI and STAI-Y, we observed that a higher BMI predicted lower subjective health perception, and lower subjective health predicted higher anxiety scores (STAI-Y). A significant indirect effect was found for BMI through subjective health on STAI-Y scores, *b* = 1.40, β = .11, *p* < .001. Concerning object valuation as a mediator between BMI and STAI-Y, we observed that higher BMI predicted a lower object valuation score, and a higher object valuation score predicted higher anxiety scores. A significant indirect effect was found for BMI through object valuation on STAI-Y scores, *b* = -0.28, β = -.02, *p* = .049.

Concerning comorbidities on STAI-Y, mediation results must be considered as *inconsistent* [50] since signs of the mediated effects for both subjective health and object valuation were inconsistent with that of the direct effect of comorbidities on anxiety. Concerning Age on STAI-Y, the total and direct effects of the model were not significant, (for total effect, *b* = -0.01, β = -.01, *p* = .87). Concerning subjective health as mediator between age and STAI-Y, we observed that older age predicted higher subjective health, and higher subjective health predicted lower anxiety scores. A significant indirect effect was found for age through subjective health on STAI-Y scores, *b* = -0.05, β = -.05, *p* = .001.

Concerning object valuation as mediator between age and STAI-Y, we observed that older age predicted higher object valuation, and higher object valuation predicted higher anxiety scores. A significant indirect effect was found for age through object valuation on STAI-Y scores, *b* = 0.07, β = .007, *p* < .001. Concerning confidence in government as mediator between age and STAI-Y, an older age predicted higher confidence in government and higher confidence in government predicted lower anxiety scores. A significant indirect effect was found for age through confidence in government on STAI-Y scores, *b* = -0.03, β = -.03, *p* = .026. Concerning Surgery on STAI-Y, the total and direct effects of the model were not significant (for total effect, *b* = -1.50, β = -.05, *p* = .23. Concerning confidence in government as a mediator between surgery and STAI-Y, we observed that surgery predicted lower confidence in government, and confidence in government predicted lower anxiety scores. A significant indirect effect was found for surgery through confidence in government on STAI-Y scores, *b* = 0.02, β = .02, *p* = .036.

## General discussion

The current research set out to shed light on how some personal characteristics (e.g., BMI, bariatric surgery, comorbidities and age) and mediating factors (e.g., subjective health) impact state anxiety during the Covid-19 Pandemic.

The first main result to be mentioned is that the surveyed women showed higher state anxiety than women from the French population [51], with respectively *M* = 55.14 (*SD* = 12.66) and *M* = 34.87 (*SD* = 11.34). The second main result concerns the relationship between anxiety and body size or other personal characteristics (surgery or not, comorbidities and age) and whether or not moderating attitudinal variables are taken into account (feeling concerned by the Covid-19 pandemic or not, subjective health perception, perceived effectiveness of the proposed measures by the government). In accordance with previous results, the level of anxiety depends in part on the fact that the women think that information concerning the Covid-19 pandemic is important to them (according to hypothesis 1a), in their perceived effectiveness of the proposed measures by the government (according to hypothesis 1f) and how healthy they feel (according to hypotheses 1h). However, anxiety does not depend on personal identification (hypothesis 1b), knowledge about Covid-19 (hypothesis 1d), Lop transportation or Lop prophylaxis (hypothesis 1e) or the change in reported practices with Covid-19 (hypothesis 1g). But personal characteristics (BMI, age, comorbidities and bariatric surgery) do not directly impact the level of anxiety (respectively hypotheses 2a, 2b, 2c, 2d; unconfirmed). However, according to hypothesis 3, our results showed that those personal characteristics impact anxiety level through attitudinal variables (indirect effect) such as feeling concerned by the Covid-19 pandemic or not, subjective health perception, or perceived effectiveness of the proposed measures by the government. Thus, body size acts on the three mediating variables which, in turn, act on the anxiety score (according to hypothesis 3). For instance, and in other words, the larger the body size, the less healthy women feel, the more anxious they are.

We highlight the fact that the three mediating variables do not all have the same effect, some (low subjective health, or high DO) increase anxiety while another (high confidence in government) decreases it. Examination of the effect sizes shows that subjective health is the most influential factor, followed by object valuation and confidence in government, both half as impactful as subjective health.

Concerning subjective health (hypothesis 1h), our results are consistent with previous studies conducted prior to the current epidemic. Some highlighted the link between subjective health and anxiety in various populations. Mokrue and Acri, for example, found that subjective health and health behaviors were predictors of symptoms of anxiety among students [52]. With an overweight or obese population, Molarius et al. [53] found that Swedish participants had a higher probability of rating their health as poor compared to people of normal weight. Examining health surveys from Portugal and Switzerland [54], found that obese people rated their health significantly worse than their normal weight counterparts. This is also true for UK residents, as shown by [55]. A long-term study with a US population [55] over a 30-year period (1976–2006), confirms the above pattern for women. More recently and in relation to the Covid-19 epidemic, the study by Szabo et al. [34] revealed that individuals who perceived their health to be below average considered Covid-19 more threatening than those who perceived themselves to be in good health. Other authors [56], show that 59% of the 434 persons with obesity reported being worried about the pandemic, and 63% specifically reported being worried about their own or their relatives’ health. Note that no difference in terms of psychological profile was recorded among pre- and post-bariatric subjects, as we find in our results. Such a result is crucial because if the subjective perception of negative health improves anxiety as we observed in the present study, obese people should be encouraged in their efforts to lose weight in order to improve their psychological state and their psychological quality of life.

Secondly, concerning object valuation, we show that the larger the body size of the participants is (BMI), the less they report that information about the spread of Covid-19 variants or about the number of people affected by Covid-19 holds their attention (hypothesis 3). This lack of interest in this social object results avoiding generating a feeling of anxiety. Such results can be explained by a denial phenomenon that could be linked to the fear that this population may have felt. Let us recall that early during the pandemic, many studies reported an increased risk of developing severe complications following Covid-19 infection in patients with obesity [57]. The finding has been given wide attention by the media, possibly helping to worsen the sense of fear of those with a current or past history of excess weight. This denial is seen here as a response to conflict and stress "by refusing to acknowledge certain painful aspects of external reality or subjective experience that would be evident to others" (DSM-IV [58]). Denial is a classic defence mechanism we can assume it takes here the form of avoiding exposure to information that would increase stress.

Thirdly, we show that concerning confidence in the perceived effectiveness of the proposed measures by the government, the larger their body size is, the less confidence they have in the government, and therefore the more anxious they are (according to hypothesis 3). Perhaps this is linked to the fact that government discourse stresses the increased risk for high BMI; therefore, the process of denial as a defence mechanism would involve denial of credit to the source and hence in this case to all government discourse (and recommendations). Note that we observed that having bariatric surgery was correlated with lower confidence in government and increased anxiety (according to hypothesis 3). Regarding body size, it may be thought that the state apparatus which sometimes seems to be equated with the press seems to offer little protection for people in a situation of obesity. A Canadian qualitative study during the spread of SARS found that inconsistent information from authorities lead to individuals questioning the credibility of the information available and this affected their compliance with lock-down [59]. In France, people were given inconsistent advice about precautionary measures and insufficient information about the spread of the disease. Thus, low levels of perceived physical health, being in a Covid-19 high-risk group, and reporting a higher physical and mental burden represent a higher threat appraisal and should therefore predict a higher evaluation of the usefulness of measures and enhanced adherence [60].

One comment can be made about links between age and anxiety (see hypotheses 2b and 3). It should be noted that the age of the women interviewed is a factor to be considered in order to better understand anxiety related to the Covid-19 pandemic. We observed that an older age predicted higher subjective health, confidence in government and object valuation (i.e., report that information about the spread of Covid-19 variants or about the number of people affected by Covid-19 holds their attention). But if the first and second variables predicted lower anxiety scores, the third one predicted higher anxiety scores (according to hypothesis 3). The reference group theory can provide us with an explanatory framework. This theory assumes that subjective assessment of health depends on the individual’s comparison group. According to [61], this theory is often used to explain elderly persons’ positive health assessments. According to this perspective, elderly adults maintain positive health perceptions when confronting illness, adjusting their perceptions of health in relation to peers of their age [62], to the health of others of the same age or to their past health [63]. Cockerham, et al. claim that judgements concerning personal health by the elderly are often relative and seem to be based largely upon how they compare themselves with peers of their age, sex, and perhaps expectations others have of their health [64]. Thus, this social comparison seems to be more beneficial to older people.

### Strengths, limitations, and future directions

The current study has the strength of a good sample, including some vulnerable populations, so the mechanisms of anxiety building can be compared across different degrees of vulnerability. Most of our findings confirm the literature and seem to confirm common sense, which is a good prospect for the quality of the data. Nevertheless, the study sheds a new light on the phenomena by showing that the effects observed result in fact from the combination of indirect (mediated) mechanisms, of which some go in opposite directions. It finds what seems to be a defence mechanism by avoiding exposure to potentially stressful information, expressed as a lack of interest for such information. The study also has several limitations. Firstly, this was a convenience sample which can produce a selection bias. Moreover, anthropometric parameters were not part of the questionnaires, and correction for such possible confounders was therefore not performed. Thirdly, the study included in this review relied on self-reports of behaviour. Fourthly, this study involves only Western women. According to Zhao, et al., the frequency of anxiety disorder diagnoses is approximately double for women with obesity as compared to men with obesity [26]. Puhl and Heuer [27] argued that obesity, or being overweight, is closely linked to social discrimination and that this is particularly true for women. Accordingly, these authors attribute the higher anxiety scores of women to the greater social discrimination they experience. However, it would be interesting to replicate our study with men. In addition, it would be interesting to explore non-western cultural settings. Culturally, some countries consider extra weight as a sign of wealth and prosperity. As such the psychological perception of the impact of the extra weight on Covid-19 could be quite different and could be an interesting comparison.

Future research should focus on prospective, theory-driven studies of predictors of particular behaviours. In addition, they should seek to corroborate our findings relying on more longitudinal and experimental design, and study other populations too (e.g. men). The explaining hypothesis of the impact of denial (or information avoidance) as a defence mechanism should be investigated with qualitative methods.

## Conclusions

Identifying obesity as a potential risk factor for anxiety disorders is crucial, not only to further our knowledge regarding this prevalent mental disorder, but also to alleviate the resulting burden of obesity in the population. However, our results suggest obesity’s impact on state anxiety level is not direct but rather mediated by several mechanisms, meaning that beliefs and practices, subjective health perception, and confidence in government are key factors to understanding anxiety building during the Covid-19 pandemic. Thus, neglecting the role of psychosocial factors in modelling the level of anxiety in obese people would risk leading public authorities to fail to identify the most at-risk people, since fundamental indicators would be set aside. To reduce anxiety in women with obesity, it is therefore necessary to focus on psychological programs that can help them improve their perception of their health, as well as the confidence they may have in institutions, especially for younger women. The feeling of being at risk, combined with the disbelief in one’s own capacity (perceived health) or to get help from institutions, logically result in anxiety. This anxiety can be fought by reducing the dissonance in two ways: minimizing the perception of risk (e.g., by avoiding exposure to stressful information) or by improving the belief that illness can be fought successfully. Public policies should rather encourage the second approach, by objectively and subjectively improving health status and the efficacy of institutions. Failing to do so may result in producing an information avoidance effect, which in turn is not good in a pandemic. Interestingly, in social media appeared some similar phenomena of denial or fantasy treatments which aim at solving the same dissonance between perceived risk and perceived self-efficacy or institutional efficacy. In short, this work is useful for psychological science and public policies in evidencing the mediator variables that explain state anxiety. Although our study is centred on a limited, especially sensitive and vulnerable population, it is likely that some mechanisms highlighted here are generic, and special attention should be given to defence mechanisms.

## Supporting information

S1 File (DOCX)

S1 Data (XLS)
