# Supplementary material for: Mediating factors explain anxiety experienced by women with obesity during the Covid-19 pandemic
Source: PLoS One. 2023 Dec 20;18(12):e0295034. doi: 10.1371/journal.pone.0295034 (PMC10732381; doi:10.1371/journal.pone.0295034)
Supplement: S1 File — (DOCX) [file pone.0295034.s001.docx]

**Appendix A: 40 items DO Questionnaire**

*Inversed items

**Involvement**

**Object Valuation**

*The extent of contamination by the Covid virus is not information that holds my attention

Information on the spread of Covid-19 variants catches my attention

*Compliance with Covid barrier measures is not important for me.

Information on the number of people suffering from Covid-19 is important for me

The reliability of the vaccine is important for me.

**Personal identification**

*I do not think that I am a person at risk of Covid-19

*I do not think that I am concerned by the Covid epidemic

I think that I am concerned by the new Covid variants

I think that I could contract a severe form of Covid-19

I think I am concerned by the respect of barrier gestures

**Perceived capacity of action**

It's up to me to respect the barrier gestures to avoid Covid

It's up to me to listen to all the information on the evolution of the virus to better protect myself.

It's up to me to get vaccinated to protect myself and my loved ones.

*There's nothing I can do to protect myself from Covid variants

It's up to me to associate as little as possible with other people so as not to be contaminated by Covid.

**Subjective Knowledge**

I'm always afraid of being wrong about what I should or shouldn't do to avoid Covid.

*I'm not sure what to do to avoid Covid

I feel confident about doing the right things to avoid Covid.

Among my friends and family, I'm the person who knows the most about Covid.

Compared to others, I know a lot about Covid

*With the new Covid variants, I no longer know which barrier gestures to follow

I don't understand much about the new Covid variants

Amongst my friends, I am the person who knows best how to protect myself against Covid-

Of all the members of my family I am the best informed about Covid-19

*Among my family members, I'm not the person who knows the most about Covid vaccines.

**Level of Practices**

I avoid talking in the metro so that I don’t get infected

I avoid talking in the metro so as not to infect the other passengers or my relatives

I never watch programs about Covid

When I go home, I wash my hands before touching objects.

I watch and research Covid-related information for more than 3 hours a day

When I buy vegetables, I wash them with soap to neutralize Covid

I heat bread in an oven to neutralize Covid.

I wear an FFP2 mask when I leave the house

When I go home, I wash my clothes to avoid contamination.

*Whether it's busy or not, I do my shopping when I need/want to

I get home delivery to avoid catching Covid

I wear a mask when my family and friends visit my home

I wear a mask in the street to avoid fines

I wash my hands with soap or gel after touching my mail

*I don't pay attention to information messages urging me to get vaccinated against Covid.

**Appendix B: Hypotheses**

We hypothesized first that participants' distance to object (DO), i.e., their beliefs and practices around Covid-19, confidence in government, level of practices with Covid-19 and subjective health perception would impact their anxiety (hypotheses 1). More precisely, we predicted different effects according to each component of DO*.* Thus, the closer participants get to object valuation (i.e., the more they think that the Covid-19 pandemic is important to them), the more they experience anxiety (hypothesis 1a). Similarly, the more personal identification they feel (i.e., "I feel concerned about Covid-19"), the more anxious there will be (hypothesis 1b). On the contrary, if they think they have a good perceived capacity for action, knowledge about Covid-19 and are taking action to avoid infection (level of practice), the lower their anxiety level will be (respectively hypotheses 1c, 1d, and 1e). We also expected that confidence in government, change in reported practices with Covid-19 and subjective health would have a positive impact (i.e., a decrease) on anxiety (respectively hypotheses 1f, 1g, and 1h).

We also hypothesized that personal characteristics would have a positive or negative impact on anxiety depending on specific variables (hypothesis 2). More precisely, based on empirical evidence of medical risk, we expected anxiety scores to increase with BMI or age (respectively hypotheses 2a and 2b), and that women with comorbidities would exhibit higher anxiety scores (hypothesis 2c). Regarding surgery, we expected that women who had bariatric surgery would exhibit lower anxiety scores (hypothesis 2d).

Lastly, to address the question raised in the literature review, we hypothesized that the components of the distance to the object (DO), confidence in government, change in reported practices around Covid-19 and subjective health perception would mediate the relationship between anxiety level (STAI-Y) and the four antecedent variables (i.e., BMI, bariatric surgery, age, and comorbidities) (hypothesis 3). For instance, the more confidence women have in the government and the more they practice Covid-19 prevention measures, the less anxious they will feel; the more women practice Covid-19 prevention measures, the less anxious they will feel; the worse their health perception will be, the more anxious they will feel.

**Appendix C: Preliminary Analysis**

First, the sensitivity of the items of each construct was studied. Several decision criteria were selected: the skewness asymmetry coefficient and the kurtosis flattening. Item selection was made according to Kim [45], which led to the removal of five items (35 items remaining). The reliability of the questionnaire was evaluated with Cronbach’s alpha [46]. One item has been discarded in each of the object valuation, personal identification, and level of practices dimensions (respectively, α = .87, α = .71., and α = .71). No item has been removed for subjective knowledge (α = .80), but all items had to be removed for perceived capacities for action (27 items remaining).

Second, in order to rule on the structural validity of the DO index, the data were subjected to a set of confirmatory factor analyses (Hereafter CFA) testing several hypotheses according to the relevant value of the retaining fit indices (see table 3 for the computed fit indexes [47-49,60]. Some authors consider the DO questionnaire as 3 or 5 components, all related to the social object studied. However, in the present study, a five-factor model couldn’t be tested because of the absence of items related to the Capacity of Action dimension. Thus, a first model was proposed in accordance with Rouquette's theoretical model. This three-dimension model consists of involvement (object valuation and personal identification), subjective knowledge and level of practices (see table 3 for fit indexes). A second four factors model has been proposed where the original involvement dimension by Rouquette was split into sub dimensions, object valuation and personal identification. Subjective knowledge and level of practice were the two other dimensions. However, data could not be considered as adjusted to this second model, even if it accounted for a larger part of explained variance. The examination of the standardized estimates for the level of practice and subjective knowledge dimensions showed that these parameters were very low for respectively 7 and 6 items. One explanation is that some items measured different dimensions. As a consequence, a five-dimension model (model 3) was also assessed by splitting the level of practice dimension into two dimensions. The first one concerned practice on transport (hereafter LoP Transportation), the second one concerned prophylactic practices (hereafter LoP Prophylaxis). This model was correctly adjusted to the data (Table 3). The final DO questionnaire (12 items) is in Appendix E.

**Table 3. Fit indices for confirmatory factor analysis for a three, four and five-dimension model.**

| **Index** | **Original theoretical model**  **(3 F)** | **Model 2**  **(4 F)** | **Model 3**  **(5F)** | **Acceptable values** |
| --- | --- | --- | --- | --- |
| **χ^2^**  **(*df*)** | 1829.97  (272) | 260.55  (48) | 96.20  (44) |  |
| ***p*(χ^2^ )** | < .001 | < .001 | < .001 | *p* > .05 |
| ***CFI*** | .65 | .93 | .98 | > .90 |
| ***TLI*** | .62 | .90 | .97 | > .90 |
| ***SRMR*** | .10 | .09 | .03 | < .08 |
| ***RMSEA*** | .10 | .09 | .05 | < .05 to < .10 |
| ***LL*** | .10 | .08 | .03 |  |
| ***UL*** | .11 | .10 | .06 |  |
| ***AIC*** | 53547.40 | 24792.45 | 24636.10 | - |
| ***BIC*** | 53880.98 | 24972.07 | 24832.82 | - |

Third, concerning the STAI-Y, Cronbach’s alpha showed the questionnaire to reach acceptable reliability, α = .92. Concerning confidence in the perceived effectiveness of the proposed measures, Cronbach’s alpha showed the questionnaire to reach acceptable reliability, α = .72, after deleting one item; one other item was removed due to its gap to limit reliability values. Concerning change of reported practices Cronbach’s alpha showed the questionnaire to reach acceptable reliability, α = .84, but two items were removed due to their gap to limit values. The mean scores and standard deviations for each measure are reported in Table 3. The final questionnaire concerning confidence in the perceived effectiveness of the proposed measures (confidence in government, 8 items) and change of reported practices (8 items) is in Appendix E.

**Appendix D: Descriptive statistics**

| Weight Status | STAY | | Subjective Health | | Object Valuation | | Personal Identification | | Subjective Knowledge | | Level of Practice Transportation | | Level of Practice Prophylaxis | | Confidence in Government | | Change in Reported Practices | |
| --- | --- | --- | --- | --- | --- | --- | --- | --- | --- | --- | --- | --- | --- | --- | --- | --- | --- | --- |
|  | *M* | *SD* | *M* | *SD* | *M* | *SD* | *M* | *SD* | *M* | *SD* | *M* | *SD* | *M* | *SD* | *M* | *SD* | *M* | *SD* |
| UW | 48.33 | 15.23 | 5.38 | 1.12 | 5.52 | 1.65 | 3.45 | 2.36 | 4.30 | 1.93 | 4.50 | 2.02 | 4.86 | 2.04 | 4.93 | 0.95 | 3.02 | 1.08 |
| NW | 54.72 | 13.33 | 5.01 | 1.27 | 5.24 | 1.89 | 3.96 | 1.82 | 3.91 | 1.70 | 4.10 | 2.02 | 4.46 | 1.92 | 4.79 | 1.01 | 3.31 | 1.01 |
| OW | 55.10 | 11.80 | 4.56 | 1.36 | 5.46 | 1.87 | 4.80 | 1.56 | 3.73 | 1.58 | 4.48 | 2.38 | 4.97 | 1.91 | 4.41 | 1.03 | 3.60 | 1.03 |
| O1 | 54.73 | 12.31 | 4.46 | 1.34 | 5.09 | 1.61 | 4.88 | 1.63 | 3.86 | 1.61 | 4.10 | 2.16 | 4.87 | 1.94 | 4.68 | 1.23 | 3.36 | 1.14 |
| O2 | 57.29 | 11.20 | 3.95 | 1.31 | 4.69 | 1.89 | 5.45 | 1.65 | 2.97 | 1.52 | 4.22 | 2.01 | 4.62 | 2.00 | 4.17 | 1.32 | 3.59 | 1.12 |
| O3 | 56.26 | 12.68 | 3.91 | 1.60 | 5.26 | 1.74 | 5.86 | 1.58 | 3.79 | 1.72 | 4.29 | 2.15 | 5.37 | 1.71 | 4.56 | 1.20 | 3.64 | 0.98 |
| Total | 55.14 | 12.66 | 4.54 | 1.33 | 5.21 | 9.19 | 4.73 | 1.77 | 3.76 | 1.68 | 4.28 | 2.12 | 4.86 | 1.92 | 4.59 | 1.13 | 3.39 | 1.06 |

Note: UW (Underweight, BMI < 18.5); NW (Normal weight, 18.5 > BMI < 24.9); OW (Overweight, 25.0 > BMI < 29.9); O1 (Class 1 obesity, 30.0 > BMI < 34.9); O2 (Class 2 obesity, 35.0 > BMI < 39.9); O3 (Class 3 obesity, BMI 40.0 and above).

**Appendix E: Final Questionnaires (except** STAI-Y**)**

*Inversed items

**DO questionnaire**

**Object Valuation**

1. Information on the spread of Covid-19 variants catches my attention

2. Information on the number of people suffering from Covid-19 is important for me

**Personal identification**

3.* I do not think that I am a person at risk of Covid-19

4. I think that I could contract a severe form of Covid-19

**Subjective knowledge**

5. Amongst my friends and my family, I am the person who has the most knowledge about Covid-19

6. Compared to others, I know a lot about Covid-19

7. Amongst my friends, I am the person who knows best how to protect myself against Covid-19.

8. Of all the members of my family I am the best informed about Covid-19

**LoP Transportation**

9. I avoid talking in the metro so that I don’t get infected

10. I avoid talking in the metro so as not to infect the other passengers or my relatives

**LoP Prophylactic**

11. I wear a mask when my family and friends visit my home

12. I wash my hands with soap or gel after touching my mail

**Confidence in government questionnaire**

1. I think that we should trust the official information from the government about the health crisis

2.* I think that Covid-19 is not being contained in order to increase the profits of the pharmaceutical industry

3.* I think that we receive ambiguous messages from the government about developments in the health crisis

4.* I think that the government is exaggerating the severity of the health crisis

5.* I think that Covid-19 has been invented by governments to control us more

6. I think that if the government announces another lockdown, it’s because there is no other solution

7. I think that the measures taken by the government about the health crisis are appropriate

8.* I think that vaccines are not much use against Covid-19

**Change in reported practices questionnaire**

1. I have breathing problems when wearing a mask, so I wear it as little as possible

2. I consume a lot of poor quality food (sweets, fatty foods, alcohol, etc.) to avoid getting depressed.

3. Since the first lockdown, I snack more as it relaxes me

4. Because of all the restrictions as a result of Covid-19, I use cannabis so I can forget that I have no (or few social activities)

5. Since the pandemic, I have eaten a balanced diet in order to stay in good health

6. Since the pandemic, I have never drunk alone

7. I have changed my dietary habits since Covid-19, I eat more vegetables and fruit

8. Since the beginning of the pandemic, I have done the cooking myself in order not to be infected by Covid-19

**Subjective Health questionnaire**

1. In your opinion, what is your general state of health?
